# Supplementary material for: Prevalence and predictors of musculoskeletal injuries among gym members in Bangladesh: A nationwide cross-sectional study
Source: PLoS One. 2024 Aug 1;19(8):e0303461. doi: 10.1371/journal.pone.0303461 (PMC11293657; doi:10.1371/journal.pone.0303461)
Supplement: S1 Text — (DOCX) [file pone.0303461.s001.docx]

**Title:** Prevalence and predictors of musculoskeletal injuries among gym members in Bangladesh: A nationwide cross-sectional study

| Q.N | Question | Answer |
| --- | --- | --- |
| Section A Socio-demographic information | | |
| 1 | Age |  |
| 2 | Gender |  |
| 3 | Weight |  |
| 4 | Height |  |
| 5 | BMI |  |
| 6 | Occupation | Student  Business  Service  Homemaker  Unemployed |
| 7 | Purpose of gym joining | Losing weight  Physical fitness  Bodybuilding  Recreation |
| 8 | How many hours do you spend working out in a day? | < 1 hours  1-2 hours  >2 hours |
| 9 | What kind of exercise do you perform at the gym (answer can be multiple) | Cardio exercise  Strength training  Powerlifting  CrossFit |
| 10 | Do you perform worm up and cool down prior to exercise? | Yes  No |
| 11 | Previous history of injury during a workout | Not at all  Once  Multiple time |
| 12 | If yes causes of that particular injury (answer can be multiple) | Over-exercising  Wrong holding  Over weight lifting  Lack of workout knowledge  Fatigues  Not cautious during exercise  Inadequate information from Trainer |
| 13 | Continue workout after any injury | Yes  No |

| 14 | Do you currently have any musculoskeletal injury/pain? | | | Yes  No | |
| --- | --- | --- | --- | --- | --- |
| **If yes please locate the site of injury/ pain** | | | | | |
| 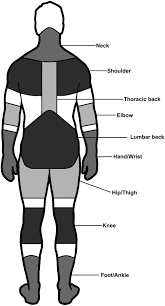 | | Trouble with the locomotive organs | | | |
|  |  | Have you had any trouble at any time during the last 7 days? | | | |
|  |  | **Neck** | Yes | |  |
|  |  |  | No | |  |
|  |  | **Shoulders** | No | |  |
|  |  |  | Right | |  |
|  |  |  | Left | |  |
|  |  |  | Both | |  |
|  |  | **Elbows** | No | |  |
|  |  |  | Right | |  |
|  |  |  | Left | |  |
|  |  |  | Both | |  |
|  |  | **Wrist/hands** | No | |  |
|  |  |  | Right | |  |
|  |  |  | Left | |  |
|  |  |  | Both | |  |
|  |  | **Upper back** | Yes | |  |
|  |  |  | No | |  |
|  |  | **Low back** | Yes | |  |
|  |  |  | No | |  |
|  |  | **Hips/thighs** | No | |  |
|  |  |  | Right | |  |
|  |  |  | Left | |  |
|  |  |  | Both | |  |
|  |  | **Knees** | No | |  |
|  |  |  | Right | |  |
|  |  |  | Left | |  |
|  |  |  | Both | |  |
|  |  | **Ankles/feet** | No | |  |
|  |  |  | Right | |  |
|  |  |  | Left | |  |
|  |  |  | Both | |  |
