# Supplementary material for: Prevalence and predictors of musculoskeletal injuries among gym members in Bangladesh: A nationwide cross-sectional study
Source: PLoS One. 2024 Aug 1;19(8):e0303461. doi: 10.1371/journal.pone.0303461 (PMC11293657; doi:10.1371/journal.pone.0303461)
Supplement: Attachment — Submitted filename: PONE-D-23-42987 with comment.pdf [file pone.0303461.s004.pdf]

PLOS ONE

Prevalence and predictors of musculoskeletal injuries among gym members in Bangladesh: A nationwide cross-sectional study.

--Manuscript Draft--

Manuscript Number:	PONE-D-23-42987
Article Type:	Research Article
Full Title:	Prevalence and predictors of musculoskeletal injuries among gym members in Bangladesh: A nationwide cross-sectional study.
Short Title:	Musculoskeletal injuries among gym members in Bangladesh
Corresponding Author:	Sohel Ahmed, BPT, MPT, MDMR<br>Ahmed Physiotherapy and Research Center<br>Dhaka, BANGLADESH
Keywords:	cross-sectional study; musculoskeletal injuries; gym-based fitness; physical fitness
Abstract:	Background: Participating in physical exercise is advantageous for maintaining optimum health, improving physical capacity, decreasing the likelihood of chronic diseases, and promoting overall wellbeing.<br>Aim: This study aimed to find out the prevalence and factors that contribute to musculoskeletal injuries among individuals who participated fitness activities at the gym.<br>Methods: Cross-sectional research was undertaken from 25th June to 30th December 2022. The survey was conducted via in-person interviews with gym members aged 18 to 50 years in Bangladesh using a structured questionnaire. The descriptive analysis facilitated the calculation of musculoskeletal pain prevalence. The gym members' predictors of musculoskeletal injuries were identified by regression analysis.<br>Results: The highest prevalence of musculoskeletal injuries at low back (36.6%) was seen among the eight body sites, followed by shoulder (24.7%) and knee (17.1%). Males (aOR 2.589, CI 1.18 to 5.65) those who go to the gym to lose weight (aOR 3.859, CI 0.91 to 16.33) and for physical fitness (aOR 1.895, CI 1.07 to 3.35) had a greater risk of musculoskeletal injury. Participants who carried out strength training exercises (aOR 4.10, CI 2.74 to 6.19) had a four-fold increased risk of musculoskeletal injuries than those who did not. Furthermore, higher adjusted odds of musculoskeletal injuries were found for the potential causes of injuries in incorrect holding (aOR 1.69, CI 1.10 to 2.60), overweight lifting (aOR 2.00, CI 1.30 to 3.08), lack of workout knowledge (aOR 3.56, CI 2.09 to 5.85), insufficient information from the trainer (aOR 5.66, CI 1.84 to 17.39).<br>Conclusion: Musculoskeletal injuries are highly prevalent among gym-goers in Bangladesh. The back was the most often injured area, followed by the shoulder and knee. It is important to exhibit caution and take extra care while doing strength training activities in order to avoid injury. Prior to engaging in gym-based activities, it is essential to have a thorough understanding of proper exercise knowledge.
Order of Authors:	Mohammad Jahirul Islam, BScPT, MPH, MDMR
	Md. Selim Rana, BPT, MPT
	Md. Sharifuddin Sarker, BPT, MDS
	Md. Mahemanul Islam, BPT
	Md. Nuruzzaman Miah, BPT, MDMR
	Md. Anwar Hossain, BPT, MDMR
	Ruwaida Jahangir, BPT
	Rahemun Akter, BPT, MPT
	Sohel Ahmed, BPT, MPT, MDMR
Opposed Reviewers:	
Additional Information:	

Powered by Editorial Manager® and ProduXion Manager® from Aries Systems Corporation

Question	Response
**Financial Disclosure**<br><br>Enter a financial disclosure statement that describes the sources of funding for the work included in this submission. Review the submission guidelines for detailed requirements. View published research articles from *PLOS ONE* for specific examples.<br><br>This statement is required for submission and **will appear in the published article** if the submission is accepted. Please make sure it is accurate.<br><br>**Funded studies**<br>Enter a statement with the following details:<br>• Initials of the authors who received each award<br>• Grant numbers awarded to each author<br>• The full name of each funder<br>• URL of each funder website<br>• Did the sponsors or funders play any role in the study design, data collection and analysis, decision to publish, or preparation of the manuscript?<br><br>Did you receive funding for this work?	The author(s) received no specific funding for this work.
**Competing Interests**<br><br>Use the instructions below to enter a competing interest statement for this submission. On behalf of all authors, disclose any competing interests that could be perceived to bias this work—acknowledging all financial support and any other relevant financial or non-financial competing interests.<br><br>This statement is required for submission and **will appear in the published article** if the submission is accepted. Please make sure it is accurate and that any funding sources listed in your Funding Information later in the submission form are also	The authors have clarified that there are no competing interests.

declared in your Financial Disclosure statement.<br><br>View published research articles from *PLOS ONE* for specific examples.<br><br>**NO authors have competing interests**<br><br>Enter: *The authors have declared that no competing interests exist.*<br><br>**Authors with competing interests**<br><br>Enter competing interest details beginning with this statement:<br><br>*I have read the journal's policy and the authors of this manuscript have the following competing interests: [insert competing interests here]*<br><br>* typeset	
**Ethics Statement**<br><br>Enter an ethics statement for this submission. This statement is required if the study involved:<br><br>• Human participants<br>• Human specimens or tissue<br>• Vertebrate animals or cephalopods<br>• Vertebrate embryos or tissues<br>• Field research<br><br>Write "N/A" if the submission does not require an ethics statement.<br><br>General guidance is provided below. Consult the submission guidelines for detailed instructions. **Make sure that all information entered here is included in the Methods section of the manuscript.**	Approved by the ethical review board of Sylhet MAG Osmani Medical College Hospital, Sylhet, Bangladesh (Approval No.: ICE/physio/22/008). Before commencing the individual interview session, written informed consent was obtained by offering a comprehensive explanation of the study's aim, objectives, potential benefits, and dangers.

**Format for specific study types**<br><br>**Human Subject Research (involving human participants and/or tissue)**<br>• Give the name of the institutional review board or ethics committee that approved the study<br>• Include the approval number and/or a statement indicating approval of this research<br>• Indicate the form of consent obtained (written/oral) or the reason that consent was not obtained (e.g. the data were analyzed anonymously)<br><br>**Animal Research (involving vertebrate animals, embryos or tissues)**<br>• Provide the name of the Institutional Animal Care and Use Committee (IACUC) or other relevant ethics board that reviewed the study protocol, and indicate whether they approved this research or granted a formal waiver of ethical approval<br>• Include an approval number if one was obtained<br>• If the study involved *non-human primates*, add *additional details* about animal welfare and steps taken to ameliorate suffering<br>• If anesthesia, euthanasia, or any kind of animal sacrifice is part of the study, include briefly which substances and/or methods were applied<br><br>**Field Research**<br><br>Include the following details if this study involves the collection of plant, animal, or other materials from a natural setting:<br>• Field permit number<br>• Name of the institution or relevant body that granted permission	
**Data Availability**<br><br>Authors are required to make all data underlying the findings described fully available, without restriction, and from the time of publication. PLOS allows rare exceptions to address legal and ethical concerns. See the PLOS Data Policy and FAQ for detailed information.	Yes - all data are fully available without restriction

A Data Availability Statement describing where the data can be found is required at submission. Your answers to this question constitute the Data Availability Statement and **will be published in the article**, if accepted.<br><br>**Important:** Stating 'data available on request from the author' is not sufficient. If your data are only available upon request, select 'No' for the first question and explain your exceptional situation in the text box.<br><br>Do the authors confirm that all data underlying the findings described in their manuscript are fully available without restriction?	
**Describe where the data may be found in full sentences. If you are copying our sample text, replace any instances of XXX with the appropriate details.**<br><br>• If the data are **held or will be held in a public repository**, include URLs, accession numbers or DOIs. If this information will only be available after acceptance, indicate this by ticking the box below. For example: *All XXX files are available from the XXX database (accession number(s) XXX, XXX.*).<br>• If the data are all contained **within the manuscript and/or Supporting Information files**, enter the following: *All relevant data are within the manuscript and its Supporting Information files.*<br>• If neither of these applies but you are able to provide **details of access elsewhere**, with or without limitations, please do so. For example:<br><br>*Data cannot be shared publicly because of [XXX]. Data are available from the XXX Institutional Data Access / Ethics Committee (contact via XXX) for researchers who meet the criteria for access to confidential data.*<br><br>*The data underlying the results presented in the study are available from (include the name of the third party*	Data will be made available prior to a request to the responding author.

Powered by Editorial Manager® and ProduXion Manager® from Aries Systems Corporation

and contact information or URL).<br>• This text is appropriate if the data are owned by a third party and authors do not have permission to share the data.<br><br>* typeset	
Additional data availability information:	

Powered by Editorial Manager® and ProduXion Manager® from Aries Systems Corporation

Cover letter

To

The Editor-in-chief,

Sir

Kindly find the enclosed original article titled, “Prevalence and predictors of musculoskeletal injuries among gym members in Bangladesh: A nationwide cross-sectional study.” for consideration for publication in your esteemed journal.

This is an extraordinary study highlighting to find out the prevalence and factors that contribute to musculoskeletal injuries among individuals who participated fitness activities at the gym. The national representative study found males those who go to the gym to lose weight and for physical fitness had a greater risk of musculoskeletal injury. Participants who carried out strength training exercises had a four-fold increased risk of musculoskeletal injuries than those who did not. Furthermore, higher adjusted odds of musculoskeletal injuries were found for the potential causes of injuries in incorrect holding, overweight lifting, lack of workout knowledge, and insufficient information from the trainer. We believe that our study makes a significant contribution to the literature.

Further, we believe that this paper will be of interest to the readership of your journal because, to date, best of our knowledge, this is the first study that addresses the serious issue.

The authors declare no conflict of interest and have no disclosures. I hereby declare that the above work is original and has not been published elsewhere, or under review by any other journal.

Thank you for your consideration. I look forward to hearing from you.

Sincerely,

Sohel Ahmed

Consultant and Founder

Ahmed Physiotherapy and Research Center, Dhaka, Bangladesh

E-mail: ptsohel@gmail.com; Phone: +88 01724007776

Manuscript

Click here to access/download;Manuscript;Manuscripy_musculoskeletal pain

Article category: Original article

Title: Prevalence and predictors of musculoskeletal injuries among gym members in Bangladesh: A nationwide cross-sectional study.

Short Title: Musculoskeletal injuries among gym members in Bangladesh

Author name(s):

Mohammad Jahirul Islam [1¶], Md. Selim Rana [2], Md. Sharifuddin Sarker [3], Md. Mahemanul Islam [4], Md. Nuruzzaman Miah [5], Md. Anwar Hossain [6], Ruwaida Jahangir [7], Rahmeun Akter [8], and Sohel Ahmed [8,9¶*]

Author(s) affiliations:

[1]Department of Physical Medicine and Rehabilitation, MAG Osmani Medical College Hospital, Sylhet, Bangladesh; physiojahir@gmail.com (M.J.I.)

[2]Department of Physiotherapy, Chittagong Institute of Medical Technology, Agrabad, Chittagong, Bangladesh; selimurr@gmail.com (M.S.R)

[3]Department of Physiotherapy, Sajida Hospital, Sajida Foundation, Dhaka, Bangladesh; physioplacebd@gmail.com (M.S.S)

[4] Department of Physiotherapy, Progetto Uomo Rishilpi International Onlus, Khulna, Bangladesh; mahamenulislamdipu@gmail.com (M.M.I)

[5]The Popular Consultation and Physiotherapy Center, Narayanganj, Dhaka, Bangladesh; drnahid00@gmail.com (M.N.M)

[6]The Barekha Hospital Madonpur Limited, Narayanganj, Dhaka, Bangladesh; anwar709@gmail.com (M.A.H)

[7]Department of Physiotherapy, Gonoshasthay Nagar Hospital, Dhaka, Bangladesh; rashedakazi5@gmail.com (R.B.J)

[8] Ahmed Physiotherapy & Research Center, Dhaka, Bangladesh; rahemun66@gmail.com (R.A)

[9] Physiotherapy Research Foundation, Dhaka, Bangladesh; ptsohel@gmail.com (S.A)

Corresponding author:

Email: ptsohel@gmail.com; (S.A)

¶ These authors contributed equally to this work.

Declarations:

Author contributions:

Conceptualization: Sohel Ahmed and Jahirul Islam

Data curation: Mohammad Jahirul Islam, Md. Selim Rana, Md. Sharifuddin Sarker, Md. Mahemanul Islam, Md. Nuruzzaman Miah, Anwar Hossain, Ruwaida Jahangir, Rahemun Akter and Sohel Ahmed

Formal analysis: Jahirul Islam, Selim Rana, and Sohel Ahmed

Investigation: Mohammad Jahirul Islam, Md. Selim Rana, Md. Sharifuddin Sarker, Md. Mahemanul Islam, Md. Nuruzzaman Miah, Anwar Hossain, Ruwaida Jahangir, Rahemun Akter, and Sohel Ahmed

Methodology: Jahirul Islam, and Sohel Ahmed

Project administration: Md. Selim Rana, Md. Sharifuddin Sarker, Md. Mahemanul Islam, Md. Nuruzzaman Miah, Anwar Hossain, Ruwaida Jahangir; and Sohel Ahmed

Resources: Mohammad Jahirul Islam, Md. Selim Rana, Md. Sharifuddin Sarker, Md. Mahemanul Islam, Md. Nuruzzaman Miah, Anwar Hossain, Ruwaida Jahangir; and Sohel Ahmed

Software: Sohel Ahmed

Supervision: Sohel Ahmed

Validation: Sohel Ahmed

Visualization: Sohel Ahmed

Writing – original draft: Sohel Ahmed and Rahemun Akter

Writing – review & editing: Sohel Ahmed and Rahemun Akter

Funding statement: This research did not receive any specific grant from funding agencies in the public, commercial or non-profit sectors.

Data availability statement: Data will be made available on request

Declaration of interests' statement: There is no potential conflict of interest to declare

Patient and public involvement: Patients and/or the public were not involved in the design, or conduct, or reporting, or dissemination plans of this research.

Patient consent for publication: Informed consent was taken from the participants prior to participating in this study.

Ethical approval: Approved by the ethical review board of Sylhet MAG Osmani Medical College Hospital, Sylhet, Bangladesh (Approval No: ICE/physio/22/008).

Title: Prevalence and predictors of musculoskeletal injuries among gym members in Bangladesh: A nationwide cross-sectional study

Abstract

Background: Participating in physical exercise is advantageous for maintaining optimum health, improving physical capacity, decreasing the likelihood of chronic diseases, and promoting overall wellbeing.

Aim: This study aimed to find out the prevalence and factors that contribute to musculoskeletal injuries among individuals who participated fitness activities at the gym.

Methods: Cross-sectional research was undertaken from 25th June to 30th December 2022. The survey was conducted via in-person interviews with gym members aged 18 to 50 years in Bangladesh using a structured questionnaire. The descriptive analysis facilitated the calculation of musculoskeletal pain prevalence. The gym members' predictors of musculoskeletal injuries were identified by regression analysis.

Results: The highest prevalence of musculoskeletal injuries at low back (36.6%) was seen among the eight body sites, followed by shoulder (24.7%) and knee (17.1%). Males (aOR 2.589, CI 1.18 to 5.65) those who go to the gym to lose weight (aOR 3.859, CI 0.91 to 16.33) and for physical fitness (aOR 1.895, CI 1.07 to 3.35) had a greater risk of musculoskeletal injury. Participants who carried out strength training exercises (aOR 4.10, CI 2.74 to 6.19) had a four-fold increased risk of musculoskeletal injuries than those who did not. Furthermore, higher adjusted odds of musculoskeletal injuries were found for the potential causes of injuries in incorrect holding (aOR 1.69, CI 1.10 to 2.60), overweight lifting (aOR 2.00, CI 1.30 to 3.08), lack of workout knowledge (aOR 3.56, CI 2.09 to 5.85), insufficient information from the trainer (aOR 5.66, CI 1.84 to 17.39).

Conclusion: Musculoskeletal injuries are highly prevalent among gym-goers in Bangladesh. The back was the most often injured area, followed by the shoulder and knee. It is important to exhibit caution and take extra care while doing strength training activities in order to avoid injury. Prior to engaging in gym-based activities, it is essential to have a thorough understanding of proper exercise knowledge.

Keywords: Cross-sectional study, musculoskeletal injuries, gym-based fitness, physical fitness

[Text]

Introduction

Engaging in physical exercise is beneficial for maintaining optimal health, enhancing physical performance, reducing the risk of chronic diseases, and enhancing overall well-being (1,2). Fitness centers serve as a vital hub for engaging in physical activity, where members tend to exercise more often than they typically do in their own homes (3,4). In 2016, the gym-based exercise participation rate among Dutch residents aged 12-79 was 21 percent, which is an increase from 12 percent in 2001 (5). Gym-based fitness activities provide a range of exercise facilities in a secure environment, promoting social engagement, and giving members access to expert's exercise instruction and coaching (3). While engaging in physical activity via sports is generally seen as advantageous, participating in fitness activities in the gym is linked to the potential for injuries (6). A descriptive epidemiological study in the Netherlands shows that many injuries occur as a result of gym-based fitness activities (6). In 2019, the total number of reported injuries resulting from fitness activities in gyms amounted to 860,000, which constituted 16% of all sports injuries in the Netherlands (7).

A research study was conducted in the Netherlands including 494 Dutch citizens who engage in exercise activities at gyms on a daily basis. Based on the survey findings, the predominant occurrence of injuries was seen during the practice of strength training. The shoulder, leg, and knee were the primary anatomical areas impacted by injury, with unsupervised gym-based exercise activities responsible for 73.1% of the reported injuries (6). Another study in South Florida revealed that out of 191 athletes, 50 individuals had a collective total of 62 injuries while participating in CrossFit over a period of 6 months (8). Research conducted in Saudi Arabia examined the occurrence of injuries among individuals who are members of gyms. The survey revealed that 29.2% of the participants reported experiencing musculoskeletal injuries. The shoulder accounted for 40.5% of injuries, followed by the foot at 32.4% and the back at 25.7% (9).

The predominant emphasis of research on exercise-related injuries in gyms has been on injuries treated in emergency departments (9), as well as injuries associated with particular exercise programs such as CrossFit (10–12), powerlifting, and weightlifting (13,14). Various fitness activities, such as CrossFit and power- and weightlifting, have undergone thorough investigation in several countries. The recent systematic reviews have collated and examined the results of these investigations (11–13). In a study conducted by Lubetzky-Vilnai A et.al., it was shown that 41.6% of the total participants (190 out of 457 persons) reported experiencing exercise-related injuries in the gym (15). The published results are limited to certain age groups, specific fitness activities performed in gyms, and injuries associated with fitness that were treated at emergency rooms. Nevertheless, these rules do not include the whole population involved in fitness activities or those specifically conducted in gyms. In order to provide appropriate guidance and education on preventing gym-related injuries, it is essential to collect extensive data on these injuries across the wider recreational exercise community. Specific data about the total number of injuries resulting

from workout activities in gyms can be obtained from countries such as The Netherlands (6) and Saudi Arab (9). Nevertheless, the comprehension of this information is restricted and there is a paucity of data regarding the prevalence and predictors of gym-related injuries. This is the first study which addressed the serious issue here in Bangladesh. The objective of this research was to determine the prevalence and factors that contribute to musculoskeletal injuries among individuals who participated fitness activities at the gyms in Bangladesh.

Materials and methods

Study design and participants

A descriptive cross-sectional study was conducted in Bangladesh from 25th June to 30th December 2022 to collect information about individuals who participate in fitness activities in gyms. The data was collected via face-to-face interviews. Due to the lack of available data on the total number of gym centers in Bangladesh and the number of individuals who regularly attend gyms, we conducted a random visit to gym facilities in five cities. In order to streamline data collection, we made a single visit to each gym facility and gathered data from the fitness trainees who were there at the time.

Ethical statement

This study adhered to the ethical standards for medical research with human participants as outlined in the World Medical Association Declaration of Helsinki (Revised 2013). A local ethical review board (Approval No: ICE/physio/22/008) approved the study protocol. The study rigorously followed the Strengthening the Reporting of Observational Studies in Epidemiology guideline for cross-sectional studies. Before commencing the individual interview session, written informed consent was obtained by offering a comprehensive explanation of the study's aim,

objectives, potential benefits, and dangers. The participants were also informed of the voluntary nature of the interview and their participation.

Subject selection criteria

The research included participants between the ages of 18 and 50, of both genders, who agreed to participate. The research eliminated those with a history of non-gym-related injuries, as well as those with musculoskeletal or neurological diseases, and participants who were using steroids.

Sample and sample size

The necessary sample size for this cross-sectional study was determined using the method for determining proportions: $n = Z\alpha^2 P (1 - P)/d^2$; where $Z\alpha = 1.96$; P = the expected musculoskeletal injuries reported in the previous literature; as we don't have any previous literature available regarding this issue, we assume 50% response distribution and d = 3% marginal error (16). Thus, the study found that a minimum sample size of 1067 was required. Based on an anticipated 10% incidence of incomplete forms, we have calculated that the absolute minimum required sample size should be 1173. A total of 1250 people were interviewed, resulting in the attainment of the final sample. Nevertheless, 44 individuals failed to respond to all the questions, so we exclude them from the final analysis.

Survey questionnaire

The questionnaire encompasses demographic data (age, gender, height, weight, BMI and occupation), workout-related details (reason for joining the gym, daily workout duration, types of exercises performed at the gym, adequacy of instructor's attention during workouts, and inclusion of warm-up/cool-down activities), injury-related information (history of previous workout-related

injuries, possible causes of injury, current pain status, continuation of workouts after an injury, adherence to rest periods following an injury, and injury management strategies).

The Nordic musculoskeletal disorder questionnaire served as a tool for pinpointing the location of pain. The questionnaire included eight distinct anatomical locations where pain was experienced, namely the neck, shoulder, elbow, wrist/hand, back, hip/thigh, knee, and ankle/foot. The Nordic musculoskeletal questionnaire's validity has been previously demonstrated in existing research (17). The questionnaire have a sensitivity for detecting pain in the body ranged from 66% to 92%, while the specificity ranged from 71% to 88% (18).

Data collection procedure

The survey was conducted using an interviewer-administered questionnaire. Eight data collectors, all of whom have graduated in physiotherapy, were selected for the purpose of data collection. The interview was conducted in Bangla. Data was gathered from five divisions of Bangladesh, where 74 fitness facilities were visited in a random manner for the purpose of data collection. Illegible participants were requested to participate in this study. Informed consent to collect, analyze, and publish their data anonymously was obtained before starting an interview. Upon receiving authorization, a data collector conducted an interview and completed a questionnaire using a paper-based format. Initially, the data collector posed a question to the respondent, and upon receiving an answer, the data collector repeated the response to the respondent in order to get confirmation.

Statistical analysis

The descriptive data were presented as frequency, percentage, mean, and standard deviation, where applicable. In order to establish the correlation between musculoskeletal pain and sociodemographic characteristics, the Pearson's Chi-square test or Fisher's exact test were used. Multiple logistic regression analysis was conducted to calculate adjusted and un-adjusted odds

ratios with a 95% confidence interval. The independent variables in this study were the presence of pain, and socio-demographic characteristics, and workout-related information as predictor variables for musculoskeletal pain. The variables were found to be statistically significant in the descriptive analysis was included into the regression model for the computation of adjusted odds ratios (aORs). The Hosmer-Lemeshow goodness-of-fit test was used to verify that the models sufficiently conform to the data. The significance level was established at <0.05. The statistical analysis was conducted using IBM SPSS Version 25.

Results

Participants' general characteristics

In total, this study examined the data of 1123 individuals. The average age, weight, height, and Body Mass Index (BMI) of the participants were 27.16 ± 6.58 years, 69.60 ± 10.67 kg, 168.59 ± 7.26 cm, and 24.28 ± 3.58 kg/m2, respectively. Table 1 presents the statistical summary of the sociodemographic characteristics. Out of the total respondents, 1039 (92.0%) were male and 538 (47.7%) belonged to the age range of 18-25 years, and 458 (40.6%) were service holder. Based on the BMI Asian classification, 442 (39.1%) of the individuals in the study were classified as obese, while 615 (54.5%) of the participants reported going to the gym to maintain their physical fitness. Out of the total participants, about 540 (47.8%) worked out for at least an hour in the gym additionally, 88.2% of the participants incorporated warm up and cooldown exercises into their routine. Of them, 67.8% did aerobic activities, 67.5% strength training, 50.0% powerlifting, and 42.8% CrossFit exercises. 25% of participants ascribed their injuries to excessive exercise, 19.9% to improper grip, 22.1% to lifting weights above their capacity, 15.3% to insufficient knowledge of exercise techniques, 5.6% to inadequate guidance from the trainer, and 16.3% to a lack of caution during exercise.

Table 1 Trainees' demographic details

Variable		Total sample (n, %)	No pain (n, %)	Presence of pain (n, %)	P-value
Total sample		1129 (100)	627 (55.5)	502 (44.5)	
**Gender**					0.008
Male		1039 (92.0)	565 (50.0)	474 (42.0)	
Female		90 (8.0)	62 (5.5)	28 (2.5)	
**Age group**					<0.001**
18-25		538 (47.7)	348 (30.8)	190 (16.8)	
26-35		493 (43.7)	246 (21.8)	247 (21.9)	
36-45		86 (7.6)	30 (2.7)	56 (5.0)	
>45		12 (1.1)	3 (0.3)	9 (0.8)	
**BMI (Asian classification)**					<0.001**
Underweight (<18.5)		23 (2.0)	16 (1.4)	7 (0.6)	
Normal weight		356 (31.5)	228 (20.8)	128 (11.3)	
Overweight		308 (27.3)	167 (14.8)	141 (12.5)	
Obese		442 (39.1)	216 (19.1)	226 (20.0)	
**Occupation**					<0.001**
Student		432 (38.3)	290 (25.7)	142 (12.6)	
Business		169 (15.0)	86 (7.6)	83 (7.4)	
Service		458 (40.6)	222 (19.7)	236 (20.9)	
Homemaker		23 (2.0)	9 (0.8)	14 (1.2)	
Unemployed		47 (4.2)	20 (1.8)	27 (2.4)	
**Purpose of GYM joining**					<0.001
Losing weight		258 (22.9)	97 (8.6)	161 (14.3)	
Physical fitness		615 (54.5)	363 (32.2)	252 (22.3)	
Bodybuilding		244 (21.6)	163 (14.4)	81 (7.2)	
Recreation		12 (1.1)	4 (0.4)	8 (0.7)	
**Hours spent working out in a day**					0.132
< 1 hour		59 (5.2)	25 (2.2)	34 (3.0)	
1 hour		540 (47.8)	298 (26.4)	242 (21.4)	
2 hours		487 (43.1)	282 (25)	205 (18.2)	
3 hours or more		43 (3.8)	22 (1.9)	21 (1.9)	
**Type of exercise performed at the gym**					
Cardio exercises	No	363 (32.2)	212 (18.8)	151 (13.4)	0.200
	Yes	766 (67.8)	415 (36.8)	351 (31.1)	
Strength training	No	367 (32.5)	148 (13.1)	219 (19.4)	<0.001
	Yes	762 (67.5)	479 (42.4)	283 (25.1)	
Powerlifting	No	565 (50.0)	292 (25.9)	273 (24.2)	0.010
	Yes	564 (50.0)	335 (29.7)	229 (20.3)	
CrossFit	No	646 (57.2)	342 (30.3)	303 (26.9)	0.042
	Yes	483 (42.8)	285 (25.2)	198 (177.5)	
**Possible causes of injury**					
Over exercise	No	847 (75.0)	507 (44.9)	340 (30.1)	<0.001

	Yes	282 (25.0)	120 (10.6)	162 (14.3)	
Wrong holding	No	904 (80.1)	541 (47.9)	363 (32.2)	<0.001
	Yes	225 (19.9)	86 (7.6)	139 (12.3)	
Overweight lifting	No	879 (77.9)	540 (47.8)	339 (30.0)	<0.001
	Yes	250 (22.1)	87 (7.7)	163 (14.4)	
Lack of workout knowledge	No	956 (84.7)	594 (52.6)	362 (33.1)	<0.001**
	Yes	173 (15.3)	33 (2.9)	140 (12.4)	
Inadequate information from the trainer	No	1066 (94.4)	623 (55.2)	443 (39.2)	<0.001**
	Yes	63 (5.6)	4 (0.4)	59 (5.2)	
Fatigueness	No	994 (88.0)	592 (52.4)	402 (35.6)	<0.001**
	Yes	135 (12.0)	35 (3.1)	100 (8.9)	
Not cautious during exercise	No	945 (83.7)	584 (51.7)	361 (32.0)	<0.001
	Yes	184 (16.3)	43 (3.8)	141 (12.5)	
**Do you perform worm up and cool down prior to exercise?**					<0.001
Yes		996 (88.2)	584 (51.7)	412 (36.5)	
No		133 (11.8)	43 (3.8)	90 (8.0)	
**Previous history of injury during a workout**					<0.001
Not at all		451 (39.9)	336 (29.8)	115 (10.2)	
Once		389 (34.5)	228 (20.2)	161 (14.3)	
Multiple time		289 (25.6)	63 (5.6)	226 (20.0)	
**Continue workout after any injury**					<0.001
Yes		554 (49.1)	274 (24.3)	280 (24.8)	
No		575 (50.9)	353 (31.3)	222 (19.7)	
**How injury managed**					<0.001
Leave it untreated		345 (30.6)	247 (21.9)	98 (8.7)	
Self-management		373 (33.0)	239 (21.2)	134 (11.9)	
Consult with physician		152 (13.5)	56 (5.0)	96 (8.5)	
Consult with physiotherapist		259 (22.9)	85 (7.5)	174 (15.4)	

Prevalence of musculoskeletal injuries

The highest prevalence of musculoskeletal injuries at low back (17.2%) was seen among the eight body sites, followed by shoulder (11.0%) and knee (7.6%). Moreover, 3.8% of the participants reported neck injuries, whereas 6.5% and 4.6% reported hip/thigh and ankle/foot injuries. Fig 1 shows the prevalence of musculoskeletal injuries among the participants. 34.5% of participants had encountered an injury at least once, whereas 25.6% of participants had experienced many instances of injuries during previous workout sessions. 49.1% of participants continued their

workout after their injury, and 63.6% of the participants left their injury untreated or managed by themselves.

Result of regression analysis

Males (aOR 2.589, CI 1.18 to 5.65) and those over 45 years of age (aOR 1.069, CI 0.19 to 6.03) had higher chances of musculoskeletal injury. Furthermore, those who go to the gym to lose weight (aOR 3.859, CI 0.91 to 16.33) and for physical fitness (aOR 1.895, CI 1.07 to 3.35) had a greater risk of musculoskeletal injury. Participants who carried out strength training exercises (aOR 4.10, CI 2.74 to 6.19) had a four-fold increased risk of musculoskeletal injuries than those who did not. Furthermore, higher adjusted odds of musculoskeletal injuries were found for the potential causes of injuries in incorrect holding (aOR 1.69, CI 1.10 to 2.60), overweight lifting (aOR 2.00, CI 1.30 to 3.08), lack of workout knowledge (aOR 3.56, CI 2.09 to 5.85), insufficient information from the trainer (aOR 5.66, CI 1.84 to 17.39), and who were not cautious during exercise (aOR 2.878, CI 1.77 to 4.66). However, those who did not do a warm-up and cool-down before to exercise had a substantially greater risk of musculoskeletal pain (aOR 2.671, CI 1.61 to 4.41). Table 2 gives the entire findings.

Table 3 Multiple logistic regression analysis: predictor of musculoskeletal injuries among the study participants

Variable	Unadjusted odd ratio	95% CI	P-value	Adjusted odd ratio	95% CI	P-value
**Gender**						
Female	Reference					**0.017**
Male	1.858	1.17 – 2.95	0.009	2.589	1.18 – 5.65	
**Age group**						
18-25	Reference					
26-35	0.182	0.05 – 0.68	0.011	0.463	0.09 -2.49	0.370
36-45	0.335	0.09 – 1.25	0.104	0.593	0.11 – 3.12	0.537
>45	0.622	0.15 – 2.47	0.500	1.069	0.19 – 6.03	0.939

**BMI**							
Underweight		Reference					
Normal weight		0.418	0.17 – 1.03	0.060	0.780	0.25 – 2.46	0.671
Overweight		0.537	0.40 – 0.71	<0.001	0.747	0.48 – 1.14	0.180
Obese		0.807	0.60 – 1.08	0.149	0.832	0.55 – 1.25	0.375
**Occupation**							
Student		Reference					
Business		0.363	0.19 – 0.67	0.001	0.349	0.15 - 0.81	0.014
Service		0.715	0.37 – 1.37	0.313	0.491	0.20 – 1.18	0.113
Homemaker		0.787	0.49 – 1.44	0.440	0.379	0.16 -0.88	0.024
Unemployed		1.152	0.41 – 3.18	0.785	0.754	0.19 – 2.94	0.684
**Purpose of GYM joining**							
Recreation		Reference					
Losing weight		4.025	1.17 – 13.76	0.026	3.859	0.91 – 16.33	0.067
Physical fitness		3.340	2.31 – 4.82	<0.001	1.895	1.07 – 3.35	0.028
Bodybuilding		1.397	1.02 – 1.90	0.035	1.010	0.63 – 1.60	0.966
**Type of exercise performed at the gym**							
Cardio exercises	Yes	0.842	0.65 – 1.08	0.182	0.798	0.53 – 1.19	0.269
	No	Reference					
Strength training	Yes	2.505	1.94 – 3.23	<0.001	4.10	2.74 – 6.19	**<0.001**
	No	Reference					
Powerlifting	Yes	1.368	1.08 – 1.73	0.009	1.38	0.92 – 2.08	0.119
	No	Reference					
CrossFit	Yes	1.279	1.01 – 1.62	0.043	1.13	0.78 – 1.66	0.498
	No	Reference					
**Possible causes of injury while workout**							
Over exercise	Yes	2.013	1.53 – 2.64	<0.001	1.28	0.86 – 1.92	0.216
	No	Reference					
Wrong holding	Yes	2.409	1.78 – 3.25	<0.001	1.69	1.10 – 2.60	**0.017**
	No	Reference					
Overweight lifting	Yes	2.984	2.22 – 4.00	<0.001	2.00	1.30 – 3.08	**0.002**
	No	Reference					
Lack of workout knowledge	Yes	6.961	4.66 – 10.39	<0.001	3.56	2.09 – 5.85	**<0.001**
	No	Reference					
Inadequate information from the trainer	Yes	20.74	7.48 – 57.52	<0.001	5.66	1.84 – 17.39	**0.002**
	No	Reference					
Fatigueness	Yes	4.208	2.80 – 6.31	<0.001	1.832	1.06 – 3.14	**0.028**
	No	Reference					
Not cautious during exercise	Yes	5.305	3.68 – 7.64	<0.001	2.878	1.77 – 4.66	**<0.001**
	No	Reference					
**Perform worm up and cool down prior to exercise?**							
No		2.967	2.02 – 4.35	<0.001	2.671	1.61 – 4.41	**<0.001**

Yes	Reference					
**Continue workout after any injury**						
Yes	1.625	1.28 – 2.06	<0.001	1.127	0.81 – 1.57	0.476
No	Reference					
**How injury managed**						
Leave it untreated	Reference					
Self-management	0.194	0.14 – 0.27	<0.001	0.184	0.11 – 0.29	<0.001
Consult with physician	0.274	0.19 – 0.38	<0.001	0.235	0.15 – 0.36	<0.001
Consult with physiotherapist	0.837	0.55 – 1.27	0.407	0.732	0.43 – 1.23	0.241

Discussion

Musculoskeletal injuries are highly prevalent among gym members in Bangladesh. Out of the eight body locations, the low back had the highest occurrence of musculoskeletal injuries, followed by the shoulder and knee. Males who work-out at the gym to improve their physical fitness and to reduce their body weight are more likely to suffer from musculoskeletal injuries. The risk of musculoskeletal injuries was four times higher for those who performed strength training activities compared to those who did not. In addition, there were increased possibilities of musculoskeletal injuries when it came to inappropriate gripping, lifting weights that are too heavy, not knowing how to work-out, and not receiving adequate advice from their trainer.

The most common site of injury was the back (17.2%), followed by the shoulder (11.0%) and the knee (7.6%). Similar to our result, we found that the study conducted by Ahmed et al. reported that back was the most commonly affected site for gym injuries, followed by the shoulder and knee. In contrast to our study, Shinde and Sahasrabuddhe reported the shoulder as the most commonly affected site for gym injuries, followed by the lower back and knee (19). The study of Feito et al. also reported that the shoulder was the most commonly affected site followed by the back, and knee (20).

Furthermore, this investigation revealed that male and those aged 45 and above had elevated susceptibility to musculoskeletal injury. Feito et al. observed a significantly higher incidence of injuries among men compared to women in their study (20). Another study found a significant difference in gym-related injuries between men and women, with men exhibiting a much higher incidence of injuries in contrast to females. However, age was not proven to be a major contributing factor (9). Nevertheless, several research studies have shown a lack of substantial disparity between males and females in terms of injury rates (21–23).

Our research found that those who participated in strength training activities had a four-fold increased probability of experiencing musculoskeletal injuries. These findings align with the data reported by Kemler et al. indicating that 52% of the participants engaged in strength training, and most of the injuries occurred during strength training activities (6). According to Gray and Finch, 55% of the injuries occurring in fitness centers and requiring treatment in emergency rooms are specifically associated with strength training. Gray and Finch also examined the precise etiology of the injuries sustained (23). They found overexertion or vigorous and/or abnormal movement emerged as a significant factor contributing to injuries in their research. Kemler et al. found that injury was most often attributed to overuse, overload, missteps and sprains, or poor posture or movement (6). Our study also found a few contributing factors, for instance, overexercise, wrong holding, overweight lifting, a lack of workout knowledge, inadequate information from the trainer, and not being cautious during exercise. Gray and Finch conducted an analysis of injuries treated in emergency departments, whereas we examined all injuries experienced by a representative sample of individuals engaged in fitness activities. This disparity in methodology might account for the variation in identified causes.

In this study, individuals who had inadequate knowledge about exercises saw a fourfold increase in injuries, while those who did not get sufficient guidance from their trainer experienced a fivefold increase in injuries. A recent review has identified several factors that can contribute to injuries during resistance training. These include overuse, insufficient rest periods after exercise, inadequate conditioning of the targeted body parts, excessive use of heavy loads, improper technique during certain exercises, and the abuse of performance-enhancing drugs (24). We firmly feel and concur that in order to avoid injuries, it is crucial to provide close monitoring and guidance on proper technique and movement during this particular activity, ideally with the assistance of a trainer. Exercises that are more intricate may need trainers with more expertise, since the trainers' experience may significantly influence the trainees' musculoskeletal discomfort. According to research conducted by Ahmed et al. (2022), fitness participants who were taught by less experienced trainers had a risk of experiencing discomfort at various locations that was more than double compared to those trained by more experienced trainers (25).

Strength and limitations

Questionnaires given by face-to-face interviewers are more accurate and have more validity as they decrease non-response and misclassification bias. The results may be more broadly applicable as a result of the random sample technique used to include the whole population which minimize the selection bias. Incorporating the nationwide population of the country may enhance the generalizability of the results. However, there are also a number of limitations to this research. First off, the reason for the correlation between the independent and dependent variables cannot be determined by this cross-sectional research. Second, since this research was conducted in urban regions, its findings may not apply to Bangladesh's other rural communities. Furthermore, the questionnaire relied on some inquiries that necessitated participants to recollect past occurrences,

thus resulting in recall bias. Future research should prioritize the collection of data pertaining to the characteristics of injuries and the specific treatment protocols necessary for their management.

Conclusion: Gym-goers in Bangladesh often have muscular injuries. The back was the most often injured area, followed by the shoulder and knee. Individuals of the male gender and those aged 45 and above had elevated probabilities of experiencing musculoskeletal injury. In addition, those who visit the gym with the intention of weight loss and improving physical fitness are at a higher risk of experiencing musculoskeletal injuries. Individuals who engaged in strength training activities had a risk of musculoskeletal injuries that was four times higher compared to those who did not participate in such exercises. It is important to exhibit caution and take extra care while doing strength training activities in order to avoid injury. Prior to engaging in gym-based activities, it is essential to have a thorough understanding of proper exercise knowledge.

Acknowledgement: The authors are thankful to the participants for providing the information used to conduct the study.

References:

1. Warburton DER, Nicol CW, Bredin SSD. Health benefits of physical activity: the evidence. CMAJ. 2006 Mar;174(6):801–9.

2. Heckel L, Eime R, Karg A, McDonald H, Yeomans C, O’Boyle I. A systematic review of the wellbeing benefits of being active through leisure and fitness centres. Leis Stud [Internet]. 2023; Available from: https://doi.org/10.1080/02614367.2023.2243654

3. Riseth L, Nøst TH, Nilsen TIL, Steinsbekk A. Long-term members’ use of fitness centers: a qualitative study. BMC Sport Sci Med Rehabil [Internet]. 2019;11(2). Available from: doi.org/10.1186/s13102-019-0114-z

4. Riseth L, Lund Nilsen TI, Hatlen Nøst T, Steinsbekk A. Fitness center use and subsequent achievement of exercise goals. A prospective study on long-term fitness center members. BMC Sports Sci Med Rehabil [Internet]. 2022;14(1):9. Available from: https://doi.org/10.1186/s13102-022-00400-w

5. Hover P, van Eldert P. Fitnessbranche in Nederland|2018. Kerncijfers Vraag—En Aanbodzijde, Factsheet 2019/1; Mulier Inst Utrecht, Netherlands [Internet]. 2019; Available from: www.mulierinstituut.nl

6. Kemler E, Noteboom L, van Beijsterveldt AM. Characteristics of Fitness-Related Injuries in The Netherlands: A Descriptive Epidemiological Study. Sport (Basel, Switzerland). 2022 Nov;10(12).

7. Stam C, Valkenberg H. Sportblessures in Nederland. Sportblessures in Nederland - Cijfers 2019. 2019.

8. Montalvo AM, Shaefer H, Rodriguez B, Li T, Epnere K, Myer GD. Retrospective Injury Epidemiology and Risk Factors for Injury in CrossFit. J Sports Sci Med. 2017 Mar;16(1):53–9.

9. Alnasser S, Alyamani A, AlDawod I, Almujil A. Prevalence and type of injuries among gym members in Saudi Arabia. Saudi J Sport Med. 2022;22(1):30–7.

10. Stracciolini A, Quinn B, Zwicker RL, Howell DR, Sugimoto D. Part I: Crossfit-Related Injury Characteristics Presenting to Sports Medicine Clinic. Clin J Sport Med Off J Can Acad Sport Med. 2020 Mar;30(2):102–7.

11. Ángel Rodríguez M, García-Calleja P, Terrados N, Crespo I, Del Valle M, Olmedillas H.

Injury in CrossFit®: A Systematic Review of Epidemiology and Risk Factors. Phys Sportsmed. 2022 Feb;50(1):3–10.

12. Barranco-Ruiz Y, Villa-González E, Martínez-Amat A, Da Silva-Grigoletto ME. Prevalence of Injuries in Exercise Programs Based on Crossfit(®), Cross Training and High-Intensity Functional Training Methodologies: A Systematic Review. J Hum Kinet. 2020 Jul;73:251–65.

13. Aasa U, Svartholm I, Andersson F, Berglund L. Injuries among weightlifters and powerlifters: a systematic review. Br J Sports Med [Internet]. 2017 Feb 1;51(4):211 LP – 219. Available from: http://bjsm.bmj.com/content/51/4/211.abstract

14. Keogh JWL, Winwood PW. The Epidemiology of Injuries Across the Weight-Training Sports. Sports Med. 2017 Mar;47(3):479–501.

15. Lubetzky-Vilnai A, Carmeli E, Katz-Leurer M. Prevalence of injuries among young adults in sport centers: relation to the type and pattern of activity. Scand J Med Sci Sports [Internet]. 2009 Dec 1;19(6):828–33. Available from: https://doi.org/10.1111/j.1600-0838.2008.00854.x

16. Charan J, Biswas T. How to calculate sample size for different study designs in medical research? Indian J Psychol Med. 2013 Apr;35(2):121–6.

17. Kahraman T, Genç A, Göz E. The Nordic Musculoskeletal Questionnaire: cross-cultural adaptation into Turkish assessing its psychometric properties. Disabil Rehabil. 2016 Oct 8;38(21):2153–60.

18. Ohlsson K, Attewell RG, Johnsson B, Ahlm A, Skerfving S. An assessment of neck and

upper extremity disorders by questionnaire and clinical examination. Ergonomics. 1994 May;37(5):891–7.

19. Shinde N, Sahasrabuddhe P. Prevalence of Musculoskeletal Pain and Injuries in Gym Instructors. Int J Heal Sci Res. 2021;11(4):62–7.

20. Feito Y, Burrows EK, Tabb LP. A 4-Year Analysis of the Incidence of Injuries Among CrossFit-Trained Participants. Orthop J Sport Med. 2018 Oct;6(10):2325967118803100.

21. Cheng TTJ, Mansor A, Lim YZ, Hossain Parash MT. Injury Incidence, Patterns, and Risk Factors in Functional Training Athletes in an Asian Population. Orthop J Sport Med [Internet]. 2020 Oct 1;8(10):2325967120957412. Available from: https://doi.org/10.1177/2325967120957412

22. da Costa TS, Louzada CTN, Miyashita GK, da Silva PHJ, Sungaila HYF, Lara PHS, et al. CrossFit®: Injury prevalence and main risk factors. Clinics (Sao Paulo). 2019;74:e1402.

23. Gray SE, Finch CF. The causes of injuries sustained at fitness facilities presenting to Victorian emergency departments - identifying the main culprits. Inj Epidemiol. 2015 Dec;2(1):6.

24. Bonilla DA, Cardozo LA, Vélez-Gutiérrez JM, Arévalo-Rodríguez A, Vargas-Molina S, Stout JR, et al. Exercise Selection and Common Injuries in Fitness Centers: A Systematic Integrative Review and Practical Recommendations. Int J Environ Res Public Health. 2022 Oct;19(19).

25. Ahmed S, Rashid M, Sarkar A sufian, Islam MJ, Akter R, Rahman MM, et al. Fitness trainers' educational qualification and experience and its association with their trainees'

musculoskeletal pain: A cross-sectional study. Sports. 2022 Jun 1;10(xxx):xxx.

Figure 1

Click here to access/download;Figure;Figure 1 Pain prevalance.png

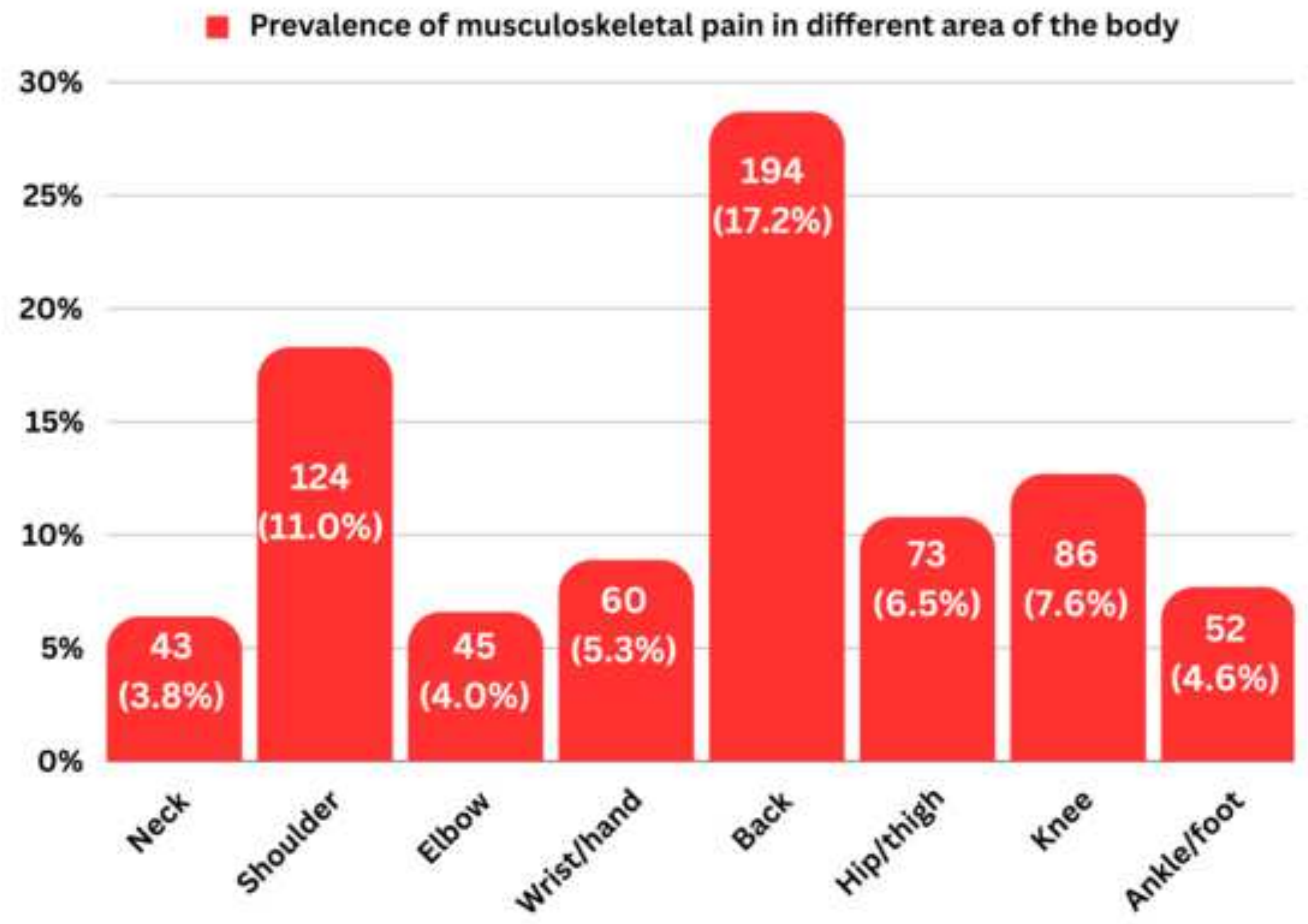

Click here to access/download
Supporting Information
S1 Text. Questionnaire_gym study.docx

Click here to access/download
Supporting Information
S2 STROBE_checklist.docx

Click here to access/download
Other
Ethical_certificate.jpg
