# Supplementary material for: Prevalence and predictors of musculoskeletal injuries among gym members in Bangladesh: A nationwide cross-sectional study
Source: PLoS One. 2024 Aug 1;19(8):e0303461. doi: 10.1371/journal.pone.0303461 (PMC11293657; doi:10.1371/journal.pone.0303461)
Supplement: Attachment — Submitted filename: Response to Reviewers.docx [file pone.0303461.s005.docx]

**Manuscript Title:** **Prevalence and predictors of musculoskeletal injuries among gym members in Bangladesh: A nationwide cross-sectional study.**

**Reply to the reviewer’s comments**

We express our gratitude to the reviewer for their meticulous and in-depth reading of this manuscript, as well as for their insightful remarks and helpful recommendations, all of which have enabled us to enhance the text's quality.

**Reviewer #1**

**Comment No: 1** It is better to report the number of participants and some statistical analytical procedure in the abstract.

**Reply No: 1** We appreciate the reviewer's insightful feedback and the chance to improve the quality of our manuscript. We have improved our manuscript in the amended text in response to your feedback, and we have marked it with YELLOW.

**Comment No: 2** Line 94: The author reported outcome from previous studies from Netherlands. However, the author needs to mention outcome from Bangladesh or similar countries (if available).

**Reply No: 2** We appreciate your suggestion; however, the altered text now includes a reference to a similar Malaysian study. There isn't a comparable published work conducted in Bangladesh.

**Comment No: 3** Line 104: The 2nd paragraph of introduction was written as discussion. In my opinion the 2nd paragraph needs to be rewritten.

**Reply No: 3** Thank you for your valuable comment. Upgraded in the revised text.

**Comment No: 4** Line 114: in third paragraph, the author again discusses the findings from Netherland and KSA. However, the needs to deduce proper rationale of the study and to focus on research gap in Bangladesh.

**Reply No: 4** Thank you for your valuable comment. Upgraded in the revised text.

**Comment No: 5** Line 143: The author needs to mention the name and institution of the review board.

**Reply No: 5** The updated manuscript now includes the name of the ethical review board.

**Comment No: 6** Line 172: The author needs to mention that the questionnaire that was used is validated in Bangladesh previously or not. If not, why they used unvalidated questionnaire.

**Reply No: 6** A popular, trustworthy, and valid instrument for evaluating musculoskeletal injuries is the Nordic Musculoskeletal Disorder Questionnaire. There isn't a previous study that evaluates Bengali language cross-cultural adaptation and validation.

**Comment No: 7** The author also needs to provide details about inclusion and exclusion criteria.

**Reply No: 7** Men and women between the ages of 18 and 50 who have agreed to participate in the study and have been regular gym goers for at least six months are included in the sample. Participants who used steroids, had musculoskeletal or neurological disorders, or a history of non-gym-related injuries were excluded from the study.

**Comment No: 8** Discussion is written proper order.

**Reply No:8** Thank you for your positive feedback.

**Reviewer #2:**

**Comment No: 1** It can be assumed that there are many cities in Bangladesh. Why did the author choose only five cities? Could you please give the names of the cities and explain why these cities were selected over others? Additionally, why did the authors exclude gyms situated in semi-urban areas or villages? Is this selection of data sources convenient? If so, how can this study be considered a nationwide survey? Please provide a definition of "nationwide" in the method section to clarify why it was included in the title. Also, are "cities" and "divisions" synonymous in the context of Bangladesh?

**Reply No: 1** We appreciate the reviewer's insightful feedback and the chance to improve the quality of our manuscript. We have improved our manuscript in the amended text in response to your feedback, and we have marked it with GREEN.

Data was collected from 74 fitness facilities from various divisional cities, districts, and gym centers at the thana level in five divisions of Bangladesh (Dhaka, Sylhet, Rajshahi, Khulna, and Chittagong). We made a random visit to gym facilities because the number of gym centers in Bangladesh and the number of people who regularly frequent gyms are not well-documented. The fact that this study was carried out in urban areas limits its applicability to other rural groups in Bangladesh. Potential samples were included using a dual-stage cluster sampling technique and a nationwide sample consisting of five administrative divisions of Bangladesh. The authors express their gratitude to the reviewer for bringing to our attention our inadvertent error with the word choice "cities." To be clear, we gathered information from 74 fitness centers spread over five administrative divisions, including metropolitan and semi-urban areas.

**Comment No: 2** Furthermore, why did the authors choose to withhold the name of the ethical review board? Is this board independent? Please provide details of the ethical approval process and consider including a copy of the approval as a supplementary file with the manuscript.

**Reply No: 2** Blinded peer review required many journals; therefore, we prepared it appropriately. We also submitted the ethical approval letter as an additional file with the submission, and we have now included the details in the updated text.

**Comment No: 3** The results suggest that 22.9% of injured patients consulted with physiotherapists, a percentage surprisingly higher than that of physicians. However, this finding may not be realistic in the context of Bangladesh. Readers may question the validity of these results, necessitating a thorough examination of the data collection methods for potential biases.

**Reply No: 3** We concur with the reviewer's comment. The participants in our study were individuals with a keen interest in physical activity and sports. Today, those interested in sports and physical activities are more acquainted with physiotherapy, possibly explaining the trend.

**Comment No: 4** What is the prevalence of musculoskeletal (MSK) issues in Bangladesh, and is there any evidence from previous studies to support this? No papers from Bangladesh have been cited in this study except for a self-citation. Did the authors search for MSK prevalence studies conducted in Bangladesh for reference? If such studies were not found, please mention this in the discussion section to provide context for the readers.

**Reply No: 4** Thank you for addressing this significant matter. Our study focuses on musculoskeletal injuries among gym members and may not be applicable to the entire population in Bangladesh. We have included a recent article on musculoskeletal issues among Bangladeshi people. There is a lack of literature on musculoskeletal injuries among the general population or gym members in Bangladesh.
