# Supplementary material for: Prevalence and predictors of musculoskeletal injuries among gym members in Bangladesh: A nationwide cross-sectional study
Source: PLoS One. 2024 Aug 1;19(8):e0303461. doi: 10.1371/journal.pone.0303461 (PMC11293657; doi:10.1371/journal.pone.0303461)
Supplement: Attachment — Submitted filename: Response to Reviewers.docx [file pone.0303461.s007.docx]

**Manuscript Title:** **Prevalence and predictors of musculoskeletal injuries among gym members in Bangladesh: A nationwide cross-sectional study.**

**Reply to the reviewer’s comments**

We express our gratitude to the reviewer for their meticulous and in-depth reading of this manuscript, as well as for their insightful remarks and helpful recommendations, all of which have enabled us to enhance the text's quality.

**Additional Editor Comments**

**Comment No: 1** Please incorporate the following comments you sent in email "Data were collected from all five geographic divisions of Bangladesh, and a dual-stage cluster sampling technique was used to include potential samples. We randomly chose the gym center as clusters in the first stage. Due to the lack of available data on the total number of gym centers in Bangladesh and the number of individuals who regularly attend gyms, we conducted a random visit to gym facilities in five divisional cities. In order to streamline data collection, we made a single visit to each gym facility and gathered data from the fitness trainees who were there at the time." under the sampling sub-section.

**Reply No: 1** We appreciate the insightful feedback and the chance to improve the quality of our manuscript. The information has been added in the revised text.

**Comment No: 2** Please provide description in the result section how your data met the following assumptions of the logistic regression "Basic assumptions that must be met for logistic regression include independence of errors, linearity in the logit for continuous variables, absence of multicollinearity, and lack of strongly influential outliers."

**Reply No: 2**

In the results section, we found that our data met the basic assumptions required for logistic regression analysis:

**Independence of Errors**: We evaluated the independence of error by analyzing the residuals (the discrepancies between observed and anticipated values). Our analysis revealed no significant patterns or correlations, suggesting that the assumption of error independence was satisfied.

**Linearity in the Logit for Continuous Variables**: We analyzed the correlation between the predictor factors and the log odds of the outcome using scatterplots and partial residual plots. The plots demonstrated a direct correlation between the continuous predictor variables and the log odds of the outcome This suggests that the assumption of linearity in the logit model was satisfied.

**Absence of Multicollinearity**: We assessed multicollinearity among the predictor variables using variance inflation factor (VIF) values. VIF values below 5.0 indicated that multicollinearity was not a concern, suggesting that the assumption of absence of multicollinearity was met.

**Lack of Strongly Influential Outliers**: We analyzed important outliers by using diagnostic plots such as Cook's distance and leverage plots. The plots demonstrated that there were no observations that had an excessively significant impact on the regression coefficients, confirming that the premise of the absence of very influential outliers was satisfied.

Overall, our data met the assumptions of independence of errors, linearity in the logit for continuous variables, absence of multicollinearity, and lack of strongly influential outliers, supporting the validity of our logistic regression analysis.

The following statement has been added in the revised text **“**The presence of multicollinearity among the independent variables was assessed using variance inflation factors (VIFs), with a predetermined threshold of VIF≤5.0.’’

**Reviewer #3**

We express our gratitude to the reviewer for their meticulous and comprehensive evaluation of this paper, as well as for their insightful remarks and valuable recommendations, which significantly contributed to enhancing the overall quality of this work.

**Comment No: 1** This statement contradicts the results presented in Table 1. According to Table 1, participants who went to the gym for physical fitness are less likely to experience pain, while those who went to lose weight experience pain. Does this statement refer to Table 2? How did the author correlate data in Table 1 and Table 2? I find it challenging to understand the significant information or new findings that the author intended to highlight in this study. The author should be clearer and provide more elaboration on these findings.

I suggest the author to put the Table number or Figure number on each statement in the results and discussion section to make it easier for the reader to understand.

**Reply No: 1** Thank you for your insightful comment. The statement has been modified in the revised text.

**Comment No: 2** Please put reference number for this statement.

**Reply No: 2** Reference has been added in the revised text

**Comment No: 3** I suggest the author to rewrite this paragraph. The sample size for males versus females in the current study is not comparable since the male population (n=1039) is much higher than the female population (n=90). Therefore, discussing the significance of injuries among men versus women is irrelevant.

**Reply No: 3** We agree with the reviewer's comment that our study had a majority of male participants. However, we have discussed the statistical findings of our study without regard to the number of participants.

**Comment No: 4** This statement should be elaborated further on the discussion.

**Reply No: 4** We appreciate your attention to this matter and your contribution to enhancing the quality of our text. The issue has been addressed in the first paragraph of the updated text.

**Comment No: 5** This sentence is a common knowledge and I don't think this is a significant finding that should be highlighted on the conclusion.

**Reply No: 5** Thank you for your structured comment. The following statement has been added in the revised text “Individuals without sufficient understanding of workout knowledge, who did not exercise with caution, and did not get proper guidance from their trainer, were at greater risk of musculoskeletal injury’’.
